# Hypoxia Inducible Factor 1 (HIF-1) Recruits Macrophage to Activate Pancreatic Stellate Cells in Pancreatic Ductal Adenocarcinoma

**DOI:** 10.3390/ijms17060799

**Published:** 2016-06-03

**Authors:** Na Li, Yang Li, Zengxun Li, Chongbiao Huang, Yanhui Yang, Mingxiao Lang, Junli Cao, Wenna Jiang, Yu Xu, Jie Dong, He Ren

**Affiliations:** 1 Department of Pancreatic Cancer, Key Laboratory of Cancer Prevention and Therapy, National Clinical Research Center for Cancer, Tianjin Medical University Cancer Institute and Hospital, Tianjin 300060, China; linazys@163.com (N.L.); 13516239159@163.com (Y.L.); 13516198199@163.com (Z.L.); chhuang@tmu.edu.cn (C.H.); ninoharuka@126.com (M.L.); daocaorenwx@163.com (J.C.); 18234140549@163.com (W.J.); happyxuyu@163.com (Y.X.); dong437241269@163.com (J.D.); 2 Key Laboratory of Hormones and Development (Ministry of Health), Tianjin Key Laboratory of Metabolic Diseases, Tianjin Metabolic Diseases Hospital & Tianjin Institute of Endocrinology, Tianjin Medical University, Tianjin 300070, China; yanhuiyangyyh@gmail.com

**Keywords:** PDAC, monocytes/macrophages, PSCs, HIF-1, CCL2

## Abstract

Hypoxia inducible factor 1 (HIF-1) is a transcription factor composed of two subunits, namely, HIF-1α and HIF-1β, in which HIF-1β is constitutively expressed. HIF-1 upregulates several hypoxia-responsive proteins, including angiogenesis factors, glycolysis solution enzymes, and cell survival proteins. HIF-1 is also associated with the degree of inflammation in the tumor region, but the exact mechanism remains unclear. This study aims to identify the molecular mechanism of recruiting monocytes/macrophages by HIF-1α in pancreatic ductal adenocarcinoma (PDAC) and the effects of macrophages on pancreatic stellate cells (PSCs). Immunohistochemistry (IHC) was performed for cluster of differentiation 68 (CD68), HIF-1α, and chemical chemokines 2 (CCL2). Western blot, real-time quantitative reverse transcription polymerase chain reaction (qRT-PCR), chromatin immunoprecipitation assay, and The Cancer Genome Atlas (TCGA) were used to verify the correlation between HIF-1α and CCL2 at protein and nucleic acid levels. Monocytes/macrophages were co-cultured with PSCs to observe their interaction. Samples showed significant correlation between CD68 and HIF-1α (*t*-test, *p* < 0.05). HIF-1α recruited monocytes/macrophages by promoting CCL2 secretion. Moreover, macrophages could accelerate the activation of PSCs. HIF-1α might promote inflammation and fibrosis of PDAC through CCL2 secretion, which may provide a novel target to treat PDAC patients.

## 1. Introduction

Pancreatic ductal adenocarcinoma (PDAC) is characterized by the presence of “desmoplasia”, which forms a barrier hindering the effective entry of chemotherapy drugs and promoting tumor growth. Leukocytes might account for approximately 50% of the total volume of solid tumors. Macrophages are one of the important cell subsets. In recent years, many studies have focused on the pathogenesis of PDAC inflammation and fibrosis, although the lack of oxygen is also a noteworthy feature of PDAC. To date, little research has been done on the relationship between hypoxia and macrophages.

Hypoxia is a common condition in the microenvironment of solid tumors [1]. In a hypoxic environment, hypoxia can induce chemokines to attract monocytes/macrophages, such as vascular endothelial growth factor A (VEGF-A), around the surrounding area. Different tumor microenvironments can affect the infiltration of monocytes/macrophages [2]. Macrophages in tumor tissues have their specific sources; tumor-derived chemokines and corresponding macrophage receptors play a critical role in the recruitment of monocytes/macrophages, such as C-C chemokine receptor type 2(CCR2)/ chemical chemokine 2 (CCL2), vascular endothelial growth factor receptor 1 VEGFR1/VEGF-A, L-selectin (CD62L)/CD62L ligands, and chemokine (C-X3-C motif) receptor 1 (CX3CR1)/ chemokine (C-X3-C motif) ligand 1 (CX3CL1). CCL2 is a potent cytokine in the recruitment of monocytes/macrophages to inflammation, tissue damage, and tumor locations; CCL2 is also known as the monocyte chemotactic protein-1 or small inducible cytokine 2. CCL2 is expressed in ovarian carcinoma, cervical squamous-cell carcinoma, melanoma, and other tumors; furthermore, CCL2 is proportional to the infiltration of monocytes/macrophages [3,4,5].

Tumor stroma is a dynamic environment, which contains inflammatory cells, pancreatic stellate cells, endothelial cells, extracellular matrix, and different growth factors and cytokines; these cells interact with tumor cells to modify their behavior. In this study, we aimed (i) to analyze whether the expression of Hypoxia inducible factor (HIF)-1α/CCL2 and HIF-1α/CD68 has a correlation from the tissue; (ii) to investigate the regulation of HIF-1α on CCL2 was verified at the level of protein and nucleic acid in cells; and (iii) to investigate the recruitment of monocytes/macrophages and the activation of pancreatic stellate cells (PSCs) *in vitro*.

Little research has been done on PDAC hypoxia, inflammation, and fibrosis. The present study reports for the first time that HIF-1α promotes the PDAC secretion of CCL2 to recruit monocytes/macrophages, and to activate PSCs. This result will provide a new idea for inflammation and fibrosis formation.

## 2. Results

### 2.1. Cluster of Differentiation 68 (CD68) Expression Was Related to Hypoxia Inducible Factor (HIF)-1α in the Samples of Pancreatic Ductal Adenocarcinoma (PDAC) Through Immunohistochemistry (IHC) Staining

**Figure 1:**
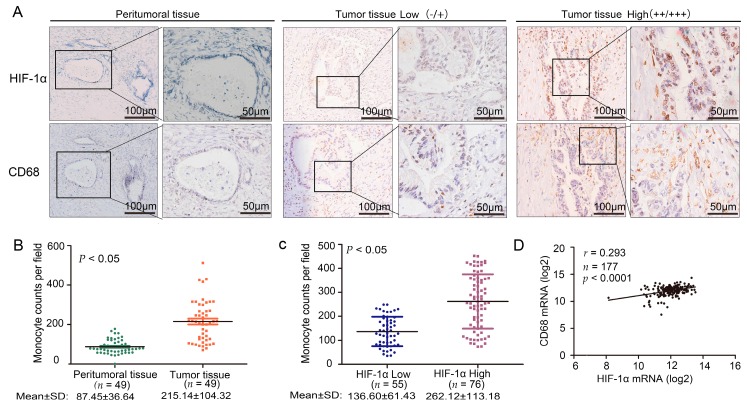
The correlation between hypoxia inducible factor (HIF)-1α and CD68 expression in specimens of pancreatic ductal adenocarcinoma (PDAC). (**A**) Immunohistochemical analysis of HIF-1α and CD68 correlative expression in consecutive sections from human PDAC surgical samples (tumor tissue and peritumoral tissue). **Left**, low expression (intensity grade − and +); **Right**, high expression (intensity grade ++ and +++) (magnification, 100 or 200); (**B**) Statistical analysis of CD68 and HIF-1α in tumor tissue and peritumoral tissue; (**C**) Statistical analysis of CD68 and HIF-1α in the PDAC surgical samples; (**D**) Analyzed messenger RNA (mRNA) expression profiles of HIF-1α and CD68 in 177 PDAC patients from The Cancer Genome Atlas (TCGA).

We used immunohistochemistry (IHC) staining to detect the expression of HIF-1α and CD68 in PDAC. We used the peritumoral tissue as a control group (Figure 1A). The number of macrophages infiltrated with tumor tissue and peritumoral tissue was statistically significant (*t*-test, *p* < 0.05) (Figure 1B). A *t*-test further confirmed that the expression levels of HIF-1α and CD68 were statistically significant (*t*-test, *p* < 0.05) (Figure 1C). We further analyzed the correlation between CD68 and HIF-1α at the mRNA level in The Cancer Genome Atlas (TCGA) (*r* = 0.293, *p* < 0.0001) (Figure 1D). Hypoxia and desmoplasia are the two major characteristics of PDAC. To obtain a positive relationship between them in PDAC, we collected 131 cases of PDAC from the Tianjin Tumor Hospital; these cases had not received any antitumor therapy. We used IHC staining to detect the expression of HIF-1α, CD68, and collagen I in PDAC (Supplementary Figure S1A). With high HIF-1α expression, the degree of fibrosis was substantially correlated with the number of monocytes/macrophages. A *t*-test confirmed that the expression levels of HIF-1α and collagen I were statistically significant (*t*-test, *p* < 0.05) (Supplementary Figure S1B).

### 2.2. HIF-1α Expression Was Significantly Correlated with Chemical Chemokines 2 (CCL2) at the Tissue Level, and CCL2 Expression Was Related to Patient Prognosis

**Figure 2:**
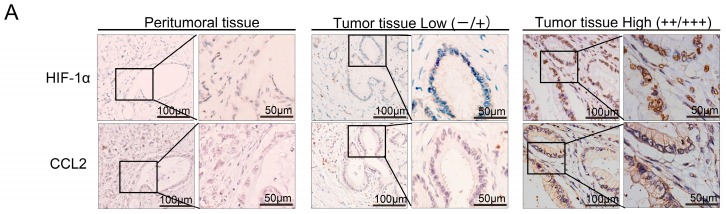

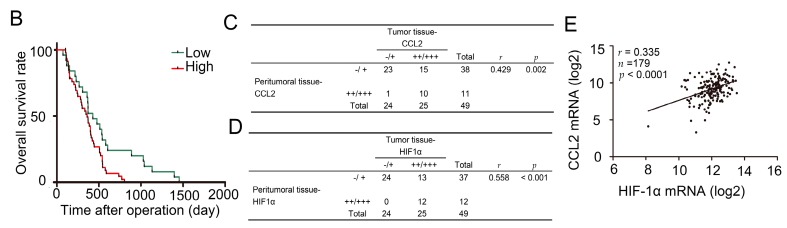
The correlation between HIF-1α and chemical chemokines 2 (CCL2) expression in specimens of PDAC. (**A**) Immunohistochemical analysis of HIF-1α and CCL2 correlative expression in consecutive sections from human PDAC surgical samples (tumor tissue and peritumoral tissue). Left, low expression (intensity grade − and +). Right, high expression (intensity grade ++ and +++) (magnification, 100 or 200); (**B**) Association between CCL2 expression levels and the overall survival of patients with PDAC. Patients with PDAC (131) were stratified into two groups according to CCL2 IHC staining intensity. Patients with high CCL2 expression (intensity grade ++ and +++) had much worse overall survival when compared with patients with low CCL2 expression (intensity grade − and +). *p* = 0.014 was determined with a log-rank test; (**C**) Statistical analysis of immunohistochemical results of CCL2 expression in tumor tissue and peritumoral tissue. *p* Value was calculated by the Spearman rank correlation test; (**D**) Statistical analysis of immunohistochemical results of HIF-1α and CCL2 expression in 131 human PDAC surgical samples. *p* Value was calculated by the Spearman rank correlation test; (**E**) Analyzed mRNA expression profiles of HIF-1α and CCL2 in 177 PDAC patients from TCGA.

We used IHC staining to detect the expression of HIF-1α and CCL2 in PDAC tissues (Figure 2A). Overall, survival of PDAC patients with low CCL2 expression was significantly longer than that of patients with high CCL2 staining (*p* = 0.014) (Figure 2B). The expression levels of CCL2 in peritumoral tissue and tumor tissue were statistically significant (*r* = 0.429, *p* < 0.05). The final staining scores of HIF-1α and CCL2 were strongly correlated with each other (*r* = 0.268, *p* < 0.05) (Figure 2C). We further analyzed the correlation between CCL2 and HIF-1α at the mRNA level in The Cancer Genome Atlas (TCGA) (*r* = 0.335, *p* < 0.0001) (Figure 2D). We also evaluated the correlation between the CCL2 expression levels and clinicopathological features (Supplementary Table S1). No evident correlation was found between the expression of CCL2 and the age, gender, and lymph node metastasis of the patients. However, CCL2 expression was related to tumor differentiation (*r* = 0.237, *p* < 0.05). Thus, CCL2 played a major role in the development of PDAC.

### 2.3. HIF-1α Regulated CCL2

**Figure 3:**
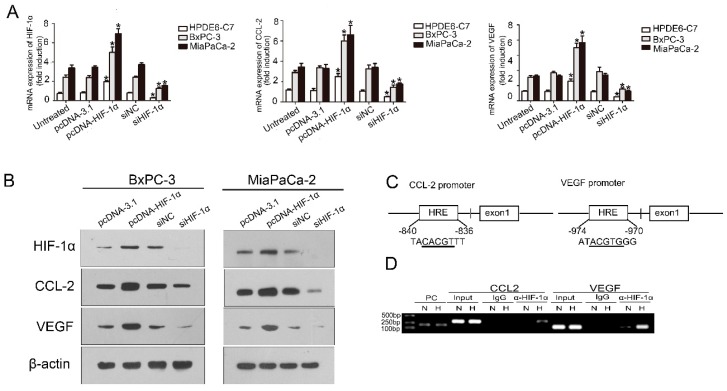
HIF-1α regulated the expression of CCL2 in PDAC cells, PC, N and H indicate positive control, normoxia, and hypoxia, respectively. (**A**) HPDE6-C7,BxPC3 and MiaPaca-2 cells were transfected with siHIF-1α (50 nmol/L) and pcDNA3.1-HIF-1α plasmids (4 µg) for 48 h, respectively; the mRNA expression levels of HIF-1α and CCL2 were assessed by qRT-PCR. The experiments were performed thrice independently. * *p* < 0.05 *vs.* control. Cells without treatment and normal pancreatic cell with transfection treatment were adopted as two negative controls. Vascular endothelial growth factor (VEGF) was used as positive control; (**B**) Western blot analysis confirmed the protein level expression correlation between HIF-1α and CCL2 in PDAC cell lines. The experiments were performed thrice independently. VEGF was used as positive control; (**C**) The DNA sequence of the CCL2 promoter (**left**) and the DNA sequence of the VEGF promoter (**right**); (**D**) Chromatin immunoprecipitation analysis in MiaPaCa-2 cells. The PCR products of VEGF promoter were used as positive control. PC, N and H indicate positive control, normoxia, and hypoxia, respectively.

CCL2 regulates the infiltration of monocytes/macrophages and is reflected in tumor sites; in addition, HIF-1α is a critical transcriptional factor. To determine whether HIF-1α regulates the transcription of CCL2 in PDAC cell lines, we used small interfering RNAs (siRNAs) targeting HIF-1α to reduce the HIF-1α expression. The CCL2 mRNA expression on the two PDAC cell lines (BxPC-3 and MiaPaCa-2) and one normal pancreatic cell (HPDE6-C7) was detected by real-time quantitative reverse transcription polymerase chain reaction (qRT-PCR). We also found that the CCL2 expression decreased. When HIF-1α was overexpressed, the CCL2 mRNA expression markedly increased (Figure 3A). Cells without treatment and normal pancreatic cell with transfection treatment were adopted as two negative controls. We used Western-blot in the two PDAC cell lines to confirm whether HIF-1α regulates CCL2 at the protein level (Figure 3B). Semenza *et al.* report that hypoxia-response elements (HRE) consist of contiguous transcription factor binding sites, which contain the core sequence of 5′-A(G)CGTG-3′; HIF-1α can activate the transcription of vascular endothelial growth factor (VEGF) by the specific HRE region [6,7]. A chromatin immunoprecipitation assay further verified the HIF-1α has a direct regulatory effect on CCL2 (Figure 3C,D). VEGF acted as a positive control. HIF-1α was found to exert a regulatory effect on CCL2 in PDAC cell lines.

### 2.4. HIF-1α Significantly Promoted the Recruitment of Macrophages

**Figure 4:**
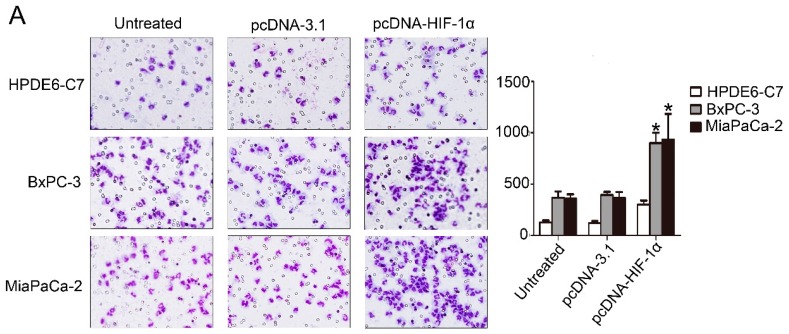

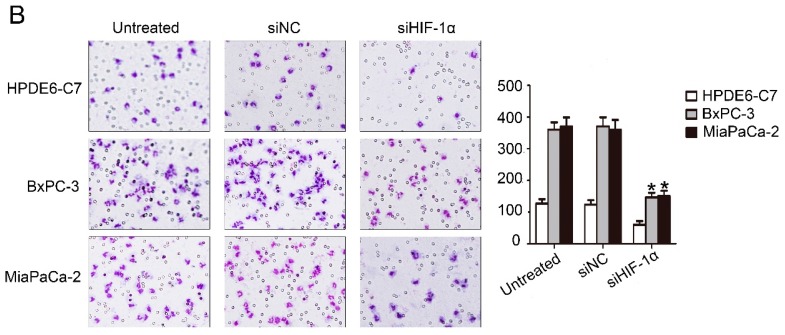
HIF-1α promoted the recruitment of macrophages. (**A**) A comparison of the ability of recruiting macrophages by HPDE6-C7, BxPC3, and MiaPaCa-2 cells transfected with pcDNA3.1 and pcDNA3.1-HIF-1α plasmids (4 µg) for 48 h using Boyden chambers. The experiments were performed thrice independently. * *p* < 0.05 *vs.* control (magnification, 100×); (**B**) A comparison of the ability of recruiting macrophages by HPDE6-C7, BxPC3, and MiaPaCa-2 cells transfected with negative control siRNA and HIF-1α siRNA (50 nmol/L) for 48 h using Boyden chambers. The experiments were performed thrice independently. * *p* < 0.05 *vs.* control (magnification, 100×).

Transwell assay was used to clarify whether HIF-1α can promote PDAC cells to recruit macrophages. The HIF-1α expression dramatically increased the recruitment of monocytes/macrophages in the two PDAC cell lines (BxPC-3 and MiaPaCa-2) and one normal pancreatic cell (HPDE6-C7) (Figure 4A). We used siRNA to knock down the expression of HIF-1α in these cell lines, and the recruitment of monocytes/macrophages significantly reduced (Figure 4B). Cells without treatment and normal pancreatic cell with transfection treatment were adopted as two negative controls.

### 2.5. Macrophages Accelerated the Activation of Pancreatic Stellate Cells (PSCs)

**Figure 5:**
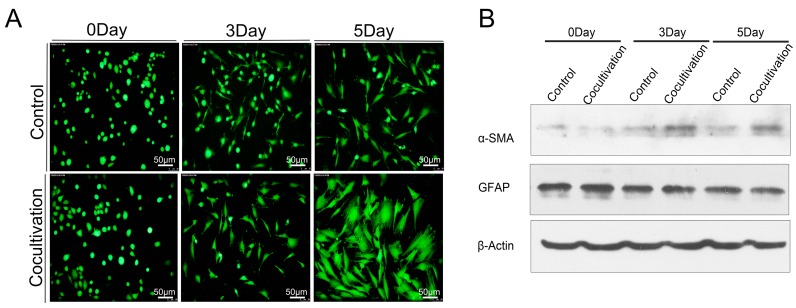

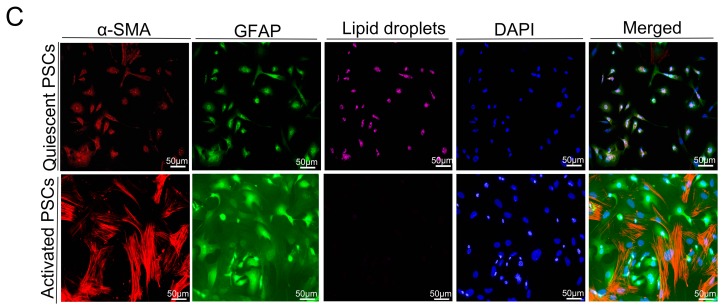
Macrophages promoted the activation of PSCs. (**A**) PSCs in co-cultured group were more active than those in the control group; (**B**) Western-blot analysis verified that the expression of α-smooth muscle actin (α-SMA) in PSCs was significantly higher than that in the control group; in addition, glial fibrillary acidic protein (GFAP) did not change significantly, and β-actin was used as a reference; (**C**) An immunofluorescence assay confirmed that the main changes in PSCs were ascribed to the disappearance of lipid droplets and the increased expression of α-SMA.

In PDACs, the fibrous stroma produced by PSCs prevents the drug from fully playing its role. Targeting PSCs is a promising way to treat PDAC [8]. The Transwell assay was utilized to explore the effect of macrophages on PSCs; the PSCs and macrophages were placed in the lower and upper chambers, respectively, to observe the activation of Calcein AM-labeled (Donjindo) PSCs. We found that macrophages could cause significant morphological changes in PSCs (Figure 5A). Western-blot analysis verified that the expression of α-SMA increased when the PSCs were activated (Figure 5B). We further confirmed the difference between quiescent and activated PSCs through immunofluorescence; the difference was the decrease of lipid droplets and the increase of α-SMA (Figure 5C). Thus, the aggravation of the inflammation in PDAC will widen the range of fibrosis, which may increase the difficulty of PDAC treatment.

## 3. Discussion

The expression level of HIF-1α in pancreatic cancer is linked to tumor progression, angiogenesis, invasion, and metastasis [9,10]. Macrophages originate mostly from the circulating mononuclear cells under the micro-environmental response. They gain different phenotypes [10,11]. Tumors in the hypoxic environment secrete numerous chemokines or other factors to promote the recruitment of monocytes/macrophages. More macrophages are present to infiltrate the hypoxia site of breast, prostate, and ovarian cancers [12]. Macrophages in tumor metastasis, progression, angiogenesis, and other processes are also important regulators [13,14,15]. The expression of CD68 and HIF-1α was studied in the samples through IHC staining. Samples showed a significant correlation between CD68 and HIF-1α (*t*-test, *p* < 0.05). PSCs and macrophages are two components playing an important role in the stroma. Hypoxia can directly increase the activity of PSCs to promote the formation of PDAC fibrosis. Interaction between PSCs and macrophages also promotes the fibrosis of pancreatic cancer. When quiescent PSCs and macrophages are co-cultured, astrocytes will be transformed into muscle fibroblasts [16]. Macrophages can produce several growth factors (transforming growth factor β1 [TGF-β1], fibroblast growth factor-2, TGF-α, and platelet-derived growth factor) and cytokines (tumor necrosis factor, interleukin-1 [IL-1], and IL-6) to activate PSCs. Activated PSCs show a myofibroblast-like phenotype. The principal changes in the PSCs include the loss of lipid droplets and the increase of α-SMA. Glial fibrillary acidic protein (GFAP) is a precise expression of intermediate filament cytoskeleton protein, which determines the structure and function of astrocytes. The expression of CD68 and collagen I was studied in the samples with high expression of HIF-1α through IHC staining. Samples with high HIF-1α expression showed a significant correlation between CD68 and collagen I (*t*-test, *p* < 0.05).

CCL2 acts as a classical chemokine that can effectively attract and activate monocytes/macrophages to local tissue damage, inflammation, tumor, and other sites. Endothelial cells, macrophages, smooth muscle cells, fibroblasts, and even tumor cells can produce CCL2 [17]. CCL2 is highly expressed in the hypoxia region, and the expression level of CCL2 depends on the severity of hypoxia [18]. The *CCL2* gene contains promoter sites associated with HIF-1α binding. Hence, the CCL2 expression can be improved by HIF-1α in a stable hypoxia environment [19]. CCL2 expression also correlates closely with HIF-1α expression in gastric cancer [20]. The expression of CCL2 and HIF-1α was studied in the samples through IHC staining; samples showed a significant correlation between CCL2 and HIF-1α (*r* = 0.268, *p* < 0.05). Overall survival of PDAC patients with low CCL2 expression was significantly longer than that of patients with high CCL2 staining. Western blot analysis, qRT-PCR, chromatin immunoprecipitation assay, and TCGA confirmed that HIF-1α exerted a regulatory effect on CCL2 in PDAC. We utilized the Transwell assay to confirm that HIF-1α was beneficial for PDAC cells to recruit monocytes/macrophages by CCL2. We co-cultured macrophages and the newly isolated PSCs. The speed of activation in the co-cultured group was significantly faster than that in the control group.

Little research has been done on PDAC hypoxia and fibrosis. The present study reported for the first time that HIF-1α promoted the PDAC secretion of CCL2 to recruit monocytes/macrophages, as well as to form PDAC fibrosis. This result provides a novel idea for fibrosis formation. Overall, these results showed that the hypoxic microenvironment of pancreatic cancer is helpful for the recruitment of local monocytes/macrophages, and macrophages can accelerate the activation of PSCs in the stroma. Fibrosis and the lack of blood supply of the interstitial hinder the survival of cancer cells and affect the delivery of chemotherapy drugs. In pancreatic cancer, fibrosis and hypoxia can lead to tumor expansion. Substantial work is needed to explore the relationships among cancer cells, macrophages, and PSCs. The effect of HIF-1α on the inflammation and fibrosis of pancreatic cancer through regulating CCL2 has been shown in the present study. Hence, HIF-1α and CCL2 are potentially important targets. Since HIF-1α mainly exists in the nucleus, and is a transcription factor, blocking it poses a certain degree of difficulty, however, CCL2 is secreted, and has a relatively specific receptor of CCR2 and CCR4 [21]. So in the hypoxia microenvironment of pancreatic cancer, blocking CCR2/CCR4 will likely have the potential to reduce the inflammation of pancreatic cancer tissue and fibrosis.

## 4. Materials and Methods

### 4.1. Immunohistochemistry

This experiment was approved by the Ethics Committee of Tianjin Medical University Cancer Hospital and Institute (Tianjin China) in 2 September 2014. The project identification code was E2014092. We collected 131 cases of PDAC from surgical specimens. IHC staining was done for HIF-1α, collagen I, CD68, and CCL2 in accordance with the manufacturers’ instructions. The staining intensity was classified as 0, 1, 2, and 3, corresponding to negative, low, medium, and high. The extent of staining was also assessed as 0, 1, 2, and 3, representing the values or range of 0%, 1%–25%, 26%–50%, and 51%–100%, respectively. The final score was the total of staining intensity and extent; each final score ranged from 0 to 9. Subsequently, the score was divided into four groups: 0, 1–2, 3–4, and 6–9, which represent the negative, low, medium, and high scores, respectively. Also, we used the symbols of −, +, ++ and+++ to form representatives of the four degrees of expression. We divided four levels of expression into two groups: low expression group (−/+) and high expression group (++/+++). All the scores were evaluated by two qualified pathological personnel.

### 4.2. Cell Culture and Hypoxic Treatment

Human PDAC cell lines (BxPC3) were purchased from the Chinese Academy of Sciences, Shanghai, China. MIAPaCa-2 cells were purchased from ATCC. The cells were grown at 37 °C in a humid atmosphere of 95% air and 5% CO_2_ and then cultured with RPMI-1640 or DMEM containing 10% fetal bovine serum (FBS). Hypoxia was produced in a sealing pot, with a debugger prepared in 94% N_2_, 5% CO_2_, and 1% O_2_ environment.

### 4.3. SiRNA Duplexes, Plasmid Constructs, and Transient Transfection

SiRNAs against HIF-1α and pcDNA-HIF-1α plasmids were used as previously described [10]. For transfection, the PDAC cells were plated in six-well plates at a density of 5 × 10^5^ cells/well. When the cells grew to 80%, about 50 nmol/L siRNAs or 4 µg of plasmids were transfected into the cells by using Lipofectamine-2000 (Invitrogen, Carlsbad, CA, USA) for 48 h.

### 4.4. Western-Blot Analysis

All cells were fully extracted using sodium dodecyl sulfate, sodium salt (SDS) cell lysis solution with protease inhibitor cocktail. Up to 20 µg of cleavage protein products were separated by sodium dodecyl sulfate polyacrylamide gel electrophoresis (SDS-PAGE), and the objective protein was detected by Western blot analysis.

### 4.5. Real-Time Quantitative Reverse Transcription Polymerase Chain Reaction (qRT-PCR)

RNA was isolated from the transfected cells by using TRIzol reagent (Invitrogen). The reverse transcription polymerase chain reaction (qRT-PCR) system (TaKaRa Bio Group, Shiga, Japan) was utilized to obtain complementary DNA (cDNA). Each sample underwent experiments in triplicate, in which β-actin acted as loading control. The PCR primers are indicated in supplementary Table S1.

### 4.6. Chromatin Immunoprecipitation Assay

A chromatin immunoprecipitation assay was performed using a commercial kit (Upstate Biotechnology, MILLIPORE, Darmstadt, Germany) according to the manufacturer’s instruction. MiaPaCa-2 was incubated under normoxia and hypoxia for 12 h and then added with 1% formaldehyde to cross link the DNA–protein complexes and lysis in SDS Lysis buffer. Lysate was required for shearing cross linked DNA to 200–1000 bp. Next, the cross linked protein was immunoprecipitated using anti-HIF-1 antibodies (Abcam, Cambridge, UK) at 4 °C overnight, the IgG was used as negative control. After washing, centrifugation, elution, and reverse, the DNA was invoked as a phenol/chloroform/isoamyl mixture and precipitated by alcohol. The DNA was used as a template for PCR of the CCL2 and VEGF binding sites (positive control). We found the promoter sequences of VEGF and CCL2 in University of California Santa Cruz (UCSC) Genome Bioinformatics Site and searched the binding sites of HIF-1α in their promoter region; then, we designed the primers with binding sites in Oligo7; finally, we used BLAST to sequence alignment. Forward and reverse primers were as follows: 5′-GGAGAATAGCTGCCATAACCA-3′, 5′-TTAAATAGCCTGCTCAAGGTC-3′ (CCL2), 5′CCTTTGGGTTTTGCCAGACTCC-3′, and 5′-TCCCTCTGACAATGTGCCATC-3′ (VEGF).

### 4.7. Isolation and Culture of Monocytes/Macrophages

This experiment was approved by the Ethics Committee of Tianjin Medical University Cancer Hospital and Institute. We performed density gradient centrifugation. Fresh peripheral blood of pancreatic cancer patients and phosphate buffered saline (PBS) were mixed with the same volume and spread above the Ficoll (Solarbio, Beijing, China). Ficoll had the equivalent volume of peripheral blood. After centrifugation (25 °C, 400× *g*, 25 min), we collected the turbid cell layer in the interface and placed it into the new tube. This layer was washed twice with PBS. When a supernatant was ruled out, cells were cultured using 1640, which contained 10% FBS. After overnight incubation, the suspended cells were deleted.

### 4.8. Transwell Assay

We used the 8 µm aperture of the 24-hole plate in this detection. Before this experiment, 2 × 10^5^ pancreatic cells were seeded onto a 6-hole plate; the next day, the cells were treated with corresponding transfection after 36 h, we collected supernatant samples of each type of cells separately and then we centrifuged them while avoiding mixed cells. These supernatant samples were injected into the low chamber, and then we placed 1 × 10^5^ macrophage cells in the upper chamber, with the culture medium of DMEM or 1640. After 24 h of incubation, macrophages were recruited to the bottom of the filter. Subsequently, a three-step method was performed (Thermo Scientific, Waltham, MA, USA). The results of each group were viewed under the microscope; we randomly selected 10 representative regions to count from which we took the average. All experiments were replicated at least thrice.

### 4.9. Isolation of PSCs

This experiment was subject to approval by the Ethics Committee of Tianjin Medical University Cancer Hospital and Institute. PSCs were isolated from the primary pancreatic cancer paraneoplastic tissues. The tissues were immersed in 3 mL of D-hank’s solution and cut into small pieces. Afterward, the tissue pieces were placed into the 37 °C digestive solution (0.05% collagenase V, 0.02% pronase, 0.03% DNase I, D-Hank’s solution, 0.5% BSA, 5 mM CaCl_2_, and 25 mM HEPES). These tissues were digested for about 20 min until no obvious tissue masses were observed. Samples were filtered, centrifuged at 450× *g* for 7 min under 4 °C, and then precipitated using a heavy suspension (0.3% BSA in D-Hank’s solution). Approximately 9.5 mL of cell suspension and 8 mL of 28.7% Nycodenz were mixed to spread under 6 mL of 0.3% BSA + D-Hank’s solution. After centrifugation (4 °C, 1400× *g*, 20 min), we collected the turbid cell layer in the interface and placed it into the new tube. This layer was washed twice with D-hank’s solution. The liquid was centrifuged at 450× *g* and 4 °C for 7 min. When the supernatant was discarded, the cells were cultured in DMEM, which contained 20% FBS.

### 4.10. Immunofluorescence

Freshly isolated PSCs were cultured on the glasses at the appropriate time the medium was drained. The PSCs were washed twice with PBS, fixed with 4% paraformaldehyde for 10 min, permeabilized with 0.1% Triton X-100 in PBS for 10 min, and then blocked for 1 h with 3% BSA in PBS. Subsequently, the cells were incubated with the antibodies α-smooth muscle actin (α-SMA) and GFAP, and DAPI was utilized to stain the nuclei. Finally, the cells were observed under a fluorescence microscope. For the lipid droplets of PSC staining, we used Nile red staining (Cell neutral lipid of Nile Red Staining Kit, GENMED, Shanghai, China). Before the start of the experiment, we took the Reagent A out of the kit, and then removed the PBS owned 2 mL buffer to the 2 mL centrifuge tube. We added 10 µL Reagent A, fully mixed it, and marked it as the working fluid. We placed it at 37 °C to avoid light incubation of cells for 10 min. At last, fluorescence cells were observed under the fluorescence microscope, and the excitation wavelength was 543 nm.

### 4.11. Statistical Analysis

Spearman’s rank correlation coefficient was utilized to test the association of ordinal variables. *t*-Test was used to compare the mean .The log-rank test was conducted to obtain a *p* value for Kaplan–Meier curves’ divergence. All probability values were two-sided. Analyses were carried out using SPSS 21.0 statistical analysis software (Armonk, NY, USA).

## 5. Conclusions

HIF-1α recruited monocytes/macrophages by promoting CCL2 secretion. Moreover, macrophages could accelerate the activation of PSCs. HIF-1α might promote inflammation and fibrosis of PDAC through CCL2 secretion, which may provide a novel target to treat PDAC patients.

## Supplementary Materials

Supplementary materials can be found at http://www.mdpi.com/1422-0067/17/6/799/s1.

## Abbreviations

PDAC	Pancreatic ductal adenocarcinoma
HIF-1	Hypoxia inducible factor 1
CCL2	Chemical chemokines 2
IHC	immunohistochemistry
TCGA	The Cancer Genome Atlas
PSCs	pancreatic stellate cells
