# Supplementary material for: Hypoxia Inducible Factor 1 (HIF-1) Recruits Macrophage to Activate Pancreatic Stellate Cells in Pancreatic Ductal Adenocarcinoma
Source: Int J Mol Sci. 2016 Jun 3;17(6):799. doi: 10.3390/ijms17060799 (PMC4926333; doi:10.3390/ijms17060799)
Supplement: Supplementary file 1 [file ijms-17-00799-s001.pdf]

Supplementary Materials: Hypoxia Inducible Factor 1 (HIF-1) Recruits Macrophage to Activate Pancreatic Stellate Cells in Pancreatic Ductal Adenocarcinoma

Na Li, Yang Li, Zengxun Li, Chongbiao Huang, Yanhui Yang, Mingxiao Lang, Junli Cao, Wenna Jiang, Yu Xu, Jie Dong and He Ren

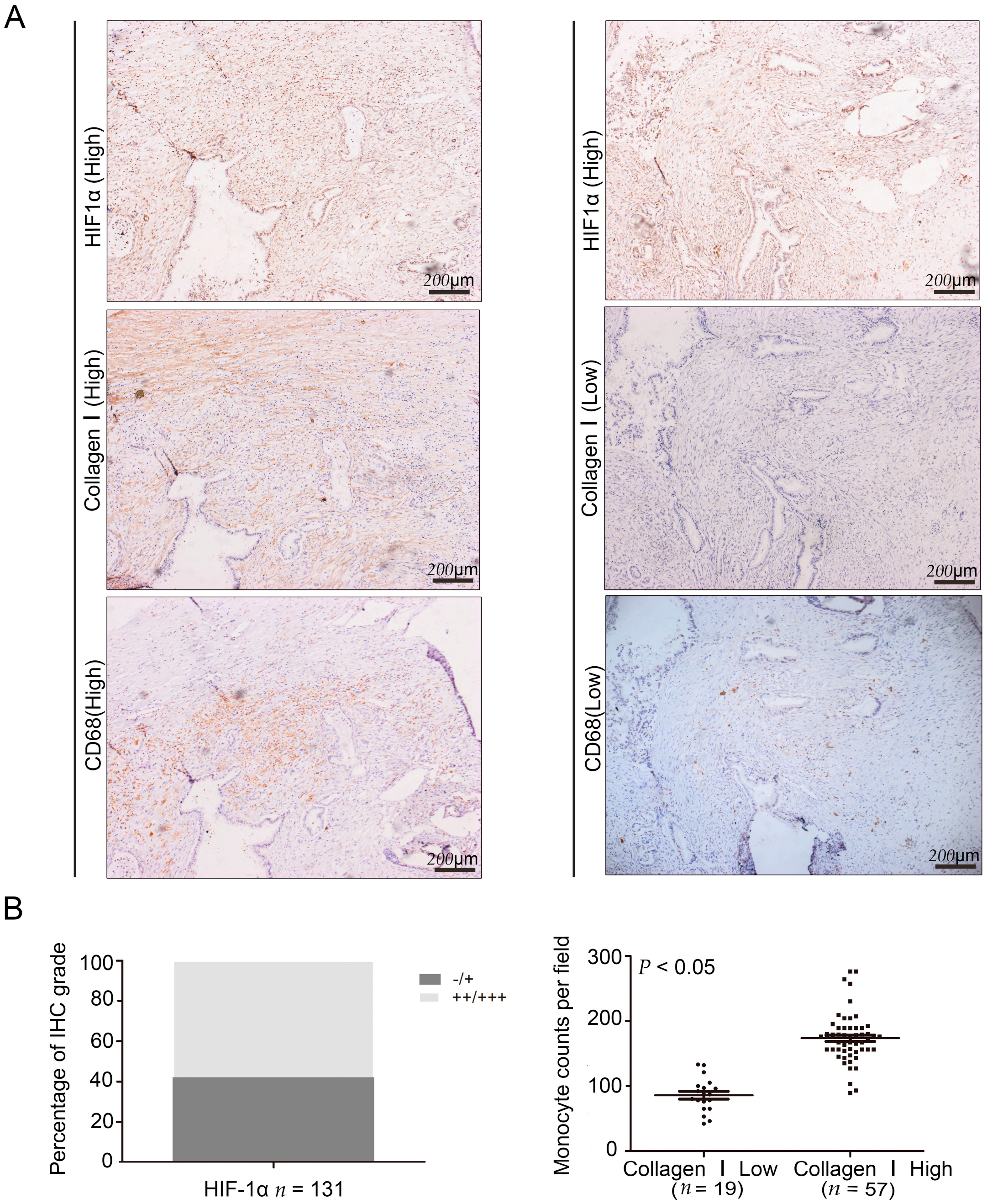

Figure S1. Expression of hypoxia inducible factor (HIF)-1α, CD68, and collagen I. Consecutive sections of pancreatic cancer tissues were prepared to detect the expression of HIF-1α, CD68, and collagen I. (**A**) Representative images for the expression of HIF-1α, CD68, and collagen I in pancreatic ductal adenocarcinoma (PDAC) tissues obtained through IHC staining; (**B**) statistical analysis of CD68 and collagen I in the PDAC surgical samples with high HIF-1α expression.

Table S1. Correlation of CCL2 expression to clinicopathological features in patients with pancreatic ductal adenocarcinoma (PDAC).

**Parameters**	**CCL2**		*r*	*p*
	**− and +**	**++ and +++**		
Sex			0.051	0.560
Male	31	46		
Female	19	35		
Age, year			0.017	0.849
<65	39	62		
≥65	11	19		
LN			0.028	0.747
−	28	43		
+	22	38		
pTNM stage			0.136	0.121
Ⅰ	9	5		
Ⅱ	12	16		
Ⅲ	16	37		
Ⅳ	13	23		
Differentiation			0.237	0.006 *
Well	23	26		
Moderate	16	14		
Poor	11	41		
Tumor size (cm)			0.068	0.438
<5	36	53		
≥5	14	28		
Blood vessel infiltration			0.026	0.768
−	37	58		
+	13	23		

p Values were obtained by using Spearman's rank correlation test, and the patients were not treated with preoperative anti-tumor therapy. LN represents local lymph node metastasis. pTNM represents the stage of tumor pathology, lymph node, and metastasis. * Represents $p < 0.05$, with statistical significance.
